# Behavioral and Neural Correlates of Executive Functioning in Musicians and Non-Musicians

**DOI:** 10.1371/journal.pone.0099868

**Published:** 2014-06-17

**Authors:** Jennifer Zuk, Christopher Benjamin, Arnold Kenyon, Nadine Gaab

**Affiliations:** 1 Laboratories of Cognitive Neuroscience, Division of Developmental Medicine, Department of Developmental Medicine, Boston Children's Hospital, Boston, Massachusetts, United States of America; 2 Harvard Medical School, Boston, Massachusetts, United States of America; 3 University of California Los Angeles, Semel Institute, Los Angeles, California, United States of America; 4 Harvard Graduate School of Education, Cambridge, Massachusetts, United States of America; University of Missouri-Kansas City, United States of America

## Abstract

Executive functions (EF) are cognitive capacities that allow for planned, controlled behavior and strongly correlate with academic abilities. Several extracurricular activities have been shown to improve EF, however, the relationship between musical training and EF remains unclear due to methodological limitations in previous studies. To explore this further, two experiments were performed; one with 30 adults with and without musical training and one with 27 musically trained and untrained children (matched for general cognitive abilities and socioeconomic variables) with a standardized EF battery. Furthermore, the neural correlates of EF skills in musically trained and untrained children were investigated using fMRI. Adult musicians compared to non-musicians showed enhanced performance on measures of cognitive flexibility, working memory, and verbal fluency. Musically trained children showed enhanced performance on measures of verbal fluency and processing speed, and significantly greater activation in pre-SMA/SMA and right VLPFC during rule representation and task-switching compared to musically untrained children. Overall, musicians show enhanced performance on several constructs of EF, and musically trained children further show heightened brain activation in traditional EF regions during task-switching. These results support the working hypothesis that musical training may promote the development and maintenance of certain EF skills, which could mediate the previously reported links between musical training and enhanced cognitive skills and academic achievement.

## Introduction

Executive functions (EF) encompass a number of cognitive processes that allow for independent and self-regulated behavior [1]. These cognitive constructs include inhibition, problem solving, goal-directed behavior, and maintenance of information in working memory [1], [2]. Another component of EF is cognitive flexibility, the ability to adjust to novel or changing task demands [3], [4], which is often captured through a task-switch design. Functional neuroimaging and repetitive transcranial magnetic stimulation (rTMS) studies have demonstrated the reliance of task-switching, or cognitive flexibility, on the prefrontal cortex (PFC) and parietal areas [5], [6], specifically the ventrolateral PFC [7], [8], [9] and the middle/medial prefrontal cortex, which encompasses the supplementary motor area (SMA) and the pre-supplementary area (pre-SMA) [10], [11], [12], [13].

The development of executive function occurs rapidly during early childhood [4], [14], though specific constructs (such as cognitive flexibility, working memory, and planning) have a long-term developmental trajectory that extends into adulthood [4], [15], [16], [17], [18], [19], [20]. Some EF constructs, such as working memory and processing speed, develop in a somewhat linear trend until early adolescence [21], [22], [23], [24], [25], [26], while others (such as rule representation and task-switching) have been suggested to follow a more specific developmental trajectory into adolescence, with increasing achievement in rule representation starting only at roughly six years of age and difficulty switching between task sets until above age nine [27], [28], [29]. Prefrontal brain regions shown to be crucial for EF are known to develop structurally throughout childhood and adolescence [30], [31], [32]. An association between reduced cortical thickness in various brain regions and enhanced performance on various EF tasks has been described in five to ten year-old children; thought to reflect selective pruning of unnecessary synaptic connections and increases in myelin [33]. Neurodevelopmental changes in cognitive flexibility have been examined through rule representation and task-switching, in which the SMA has been implicated in task-switching and the ventrolateral prefrontal cortex (VLPFC) was found to support rule representation in individuals from eight years of age to adulthood [9]. Thus, substantial evidence indicates that constructs of EF, such as cognitive flexibility and working memory, begin to develop in childhood and continue through adolescence.

Overall, EF abilities have been shown to be more predictive of academic readiness for schooling than intelligence [34] and predict math and reading skills throughout all grades [35]. Specific features of EF, cognitive flexibility and inhibitory control, demonstrate a strong relationship with mathematics and literacy skills in kindergarteners [34], [36], [37]. Evidence suggests that the executive function system is imperative for academic achievement at all grade levels [38]. Thus, EF development seems to be crucial for academic readiness and long-term achievement.

Various extra and intra-curricular activities have been shown to improve EF skills in children. For instance, Tools of the Mind, a curriculum especially designed to enhance EF skills and social/emotional development in preschool children [39], has demonstrated improved executive function abilities upon completion of the program compared to control training [40]. Extracurricular activities shown to enhance EF development in school-age children include martial arts [41], mindfulness training [42], and physical exercise [43], [44], [45]. While these findings support the potential for extracurricular activities to boost EF skills, interpretations are limited due to methodological weaknesses in these studies. Limitations include, for instance, a lack of passive or active control groups and/or the potential influence of other factors leading to improved performance, such as motivation or social engagement [42], [44]. Despite these methodological considerations, it has been suggested that EF skills can be enhanced by activities that do not solely focus on EF skills exclusively through their social, emotional, and physical engagement [46].

One extracurricular activity of recent interest to researchers is music, and its link to EF skills has been debated [47], [48], [49], [50]. Playing a musical instrument (especially within an ensemble setting) requires many sub-skills associated with EF such as sustained attention, goal-directed behavior and in particular the task-switching demands of cognitive flexibility. Individuals with musical training have demonstrated enhanced general cognitive, academic and language abilities when compared to those without musical training, and this connection may be mediated by EF [51], [52]. For example, higher intellectual functioning has been reported in children and adults with as compared to those without musical training through both cross-sectional and longitudinal study designs, though this connection remains debated [53], [54], [55], [56]. Musicians have shown enhanced language skills compared to non-musicians across several domains, namely vocabulary knowledge [57], pitch processing in speech [58], selective attention for speech in noise [59], [60], and prosody perception [61]. Perceptual abilities in the music domain have been shown to correlate with early reading skills and phonological processing in pre-readers and kindergarten-age children [62], [63]. In addition, musical training has been demonstrated to significantly relate to academic performance, specifically reading ability [64], [65], [66], [67], [68], [69] and mathematical achievement [70], [71]. Furthermore, musical training has been linked to altered brain structure and function [72], [73]. Presently, it remains unclear whether any components of EF could be contributing to these observed connections between musical training and cognitive-linguistic abilities.

To date, only a few studies have investigated the relationship between musical training and EF constructs in children and adults. Superior performance has been demonstrated in children and adults with musical training over non-musician controls on measures of auditory and visual working memory [74], [75], [76], [77], [78], [79]. Pallesen and colleagues [79] examined working memory for musical sounds in musicians and non-musicians, resulting in heightened activation for musicians compared to non-musicians in neuronal networks that sustain attention and cognitive control, which included prefrontal regions and the SMA. Furthermore, the relationship between task performance and activation pattern was stronger in musicians than in non-musicians, especially during the highest working memory loads. The authors suggest that ‘superior working memory task performance in musicians relies on an enhanced ability to exert sustained cognitive control,’ which was reflected through the hyperactivation in areas that support the processing of these constructs. However, additional components of EF beyond working memory were not considered in these studies.

For studies that have examined EF performance in trained musicians, the mixed findings reported are likely due to various methodological limitations regarding the validity of the assessments employed and subject inclusion criteria. Enhanced processing in adult musicians has been reported for components of EF, demonstrated through a nonverbal spatial task and both auditory and visual Stroop tasks [80], [81]. In addition, the hypothesis that the connection between musical training and IQ is mediated by EF has previously been proposed and tested [47], [49]. Interestingly, the findings of these cross-sectional studies diverge. One study reported significant associations between musical training and numerous EF constructs in children [49], whereas in another study no superior performance was found on any measures of EF in musically trained children compared to those without training [47]. The discrepant findings may be due to no inclusion of a control or comparisons to a control group that was not carefully screened to have no musical experience; unknown variation in the intensity and longevity of training of the musicians; or the inconsistent implementation of standardized EF measures [47], [48], [49], [50]. Differences in socioeconomic status between musicians and non-musicians may also be the source of inconsistent findings [47], [80]. Lastly, it is difficult to decipher whether these putative effects are due to musical training directly or instead a predisposition to succeed in music and higher-level cognitive tasks in general [47].

In order to address the causal nature of this hypothesized connection between musical expertise and EF abilities, the influence of musical training on EF development has also been examined longitudinally. Six months of individualized piano instruction demonstrated improved EF abilities, specifically cognitive flexibility and working memory, in elderly subjects with minimal musical experience [82]. However, this study did not employ an active control group and effects did not survive a correction for multiple comparisons so these results should be interpreted carefully. One other intervention examined kindergarten-age children following twenty days of a music-based computerized training program and demonstrated improvement in verbal intelligence and behavioral performance on a go/no-go inhibition task when compared with a control group who completed a visual arts program. This improvement correlated positively with a change in peak P2 amplitude (post-test versus pre-test) during the inhibition task in the music group only [83]. Although the study did not evaluate traditional musical training (being a computerized music program), the findings support the hypothesis that music-based intervention may play a positive role in early EF development.

It is evident that musical training relates to cognitive abilities, but it remains somewhat unclear which constructs of EF, if any, may mediate this connection. In the present study, we seek to (a) evaluate the relationship between intensive instrumental musical training and EF skills through a cross-sectional design that addresses the limiting factors of previous studies that resulted in mixed findings, and (b) compare the neural correlates of EF skills in musically trained as compared to untrained children. We assessed adults with extensive musical training and school-age musically trained children, documenting the intensity and longevity of training, and included only adult non-musicians and musically untrained children that were carefully screened to have no prior musical training beyond general curricular requirements. Several indicators of socioeconomic status were reported and matched between musicians and non-musicians, and our groups were matched for IQ to avoid any confounds of higher intelligence in the group comparisons. Further, we implemented a standardized battery of EF measures that assessed cognitive flexibility, inhibition, verbal fluency, working memory, and processing speed. In addition, this is the first study to examine the neural correlates of executive functioning, specifically task-switching (rule representation and task-set reconfiguration, adapted after Crone and colleagues [8]), in children with versus without musical training using functional magnetic resonance imaging (fMRI). We hypothesized that adults with musical training would show heightened EF abilities when compared to non-musicians, and that this difference may also be observed at a younger age in musically trained children compared to untrained children. We were especially interested to evaluate measures of cognitive flexibility and task-switching, since musical expertise involves rapid adjustments to changes in tempo, key signature, rhythm, and musical style. We hypothesized that these task-switching demands of musical training would reveal enhanced cognitive flexibility skills in those with musical training. For the neuroimaging component, we hypothesized that if musically trained children demonstrate superior EF abilities, accordingly they would show enhanced activation within prefrontal cortices when compared to non-musicians during rule representation and task-switching. In particular, we hypothesized enhanced activation in pre-SMA/SMA and VLPFC based on previous results by Crone and colleagues that demonstrated enhanced activation in these regions during rule representation and task-switching over the course of development, as well as the findings of Pallesen and colleagues which showed enhanced activation in these regions for adult musicians compared to non-musicians during a working memory paradigm. Our analysis with carefully controlled inclusion criteria and standardized measures of assessment aims to clarify the current mixed findings regarding the putative relationship between musical training and EF abilities, and explore the associated neural correlates of task-switching in musically trained compared to untrained children through fMRI.

## Methods

### Adult Participants

30 healthy, right-handed, monolingual, English-speaking adults (15 musicians (9 male, 6 female) and 15 non-musicians (9 male, 6 female), age range: 18-35 yrs, mean: 24.80 yrs; STD: 3.48 yrs) took part in the present study. Adult musicians were either seeking or had obtained a music performance degree and were working professionals. Adult musicians had commenced musical study by or before the age of 9 (mean start: 5.73 yrs, STD: 1.62 yrs), had received private lessons, were presently playing at least 8 hours per week (mean: 21.87 hrs/wk, STD: 11.49 hrs) and had studied music continuously since the onset of training. All musicians actively pursued multiple instruments while maintaining one principal instrument (type of principal instrument described in Table 1). Adult non-musicians had no musical training outside of the requirements of the general music curriculum in school.

**Table 1:** Group characteristics of musical experience in adult musicians and musically trained children.

	Mean ± SD
**Adult Musicians (n = 15)**	
***Group Characteristics***	
Age at onset of musical training (years)	5.73±1.62
Intensity of practice time/week (hours)	21.87±11.49
Duration of musical training (years)	5.2±1.33
***Type of Musical Instrument***	*Number of adults*
Piano	6
Strings	5
Woodwinds	1
Brass	2
Harp	1
**Musically Trained Children (n = 15)**	
***Group Characteristics***	
Age at onset of musical training (years)	5.86±1.41
Intensity of practice time/week (hours)	3.74±2.63
Duration of musical training (years)	5.2±1.33
***Type of Musical Instrument***	*Number of children*
Piano	5
Strings	5
Woodwinds	2
Guitar	1
Percussion	2

### Child Participants

27 children (15 musically trained (7 male, 8 female) and 12 untrained (4 male 8 female), age range: 9-12 yrs; mean 10.9 yrs, STD: 1.2 yrs) took part in this study. Musically trained children had played an instrument for a minimum of two years in regular private music lessons, started training on average at age 5 (mean: 5.86 yrs, STD: 1.41 yrs) and had been studying their instrument on average 5.2 years (STD: 1.33 yrs). More information on the details of musical training can be found in Table 1. Untrained children had no musical training outside of the requirements of the general music curriculum in school.

### General Demographics

No significant group differences in age, gender, or IQ were observed for adults or children (*p*<0.05; see Table 2). Adult participants and guardians of children completed an evaluation of current socioeconomic status (adapted from the MacArthur Research Network: http://www.macses.ucsf.edu/Default.htm). One adult musician and the guardians of four musically trained and four untrained children did not provide socioeconomic status documentation. Musicians and non-musicians in both age ranges did not differ in parent education, current job activity, or money earned in the last 12 months (all *p*>0.05; Table 2). None of the participants had a history of neurological or psychological disorder, head injuries, poor vision or hearing.

**Table 2:** Group characteristics of musicians and non-musicians in adults and children.

		Musicians	Non-musicians	P-Values	
		Mean ± SD	Mean ± SD	Mus vs. Non	
**Adults (n = 30)**					
***Group Characteristics***				**sig. 2-tailed** *Independent samples t-test*	
IQ					
WASI	Verbal Ability	63.73±5.79	61.80±7.63	0.441	
	Nonverbal Ability	60.80±6.01	57.20±5.80	0.106	
**Socioeconomic Status**		(Mean Rank)	(Mean Rank)	**sig. 2-tailed** *Mann Whitney Test*	
Adult Education		15.27	14.71	0.852	
Current job responsibility		14.13	15.93	0.524	
Parent Education		14.83	16.17	0.668	
Total Combined Family Income^b^		14.62	13.43	0.693	
***Executive Function Measures***				**sig. 1-tailed** *Independent samples t-test*	
DKEFS	Trail Making	9.00±2.39	8.80±3.19	0.423	
	Verbal Fluency	11.80±3.90	8.87±3.38	0.018	* +
	Color-Word Interference	11.27±1.10	10.73±1.79	0.160	
	Design Fluency	15.07±2.37	12.33±2.72	0.003	** +
					
WAIS	Digit Span Backwards	14.47±3.25	10.40±3.42	0.001	** +
	Coding	13.40±2.90	11.93±3.15	0.098	
**Children (n = 27)**					
***Group Characteristics***				**sig. 2-tailed** *Independent samples t-test*	
IQ					
KBIT	Non-Verbal Ability a	119.60±9.34	117.70±11.24	0.665	
**Socioeconomic Status**		(Mean Rank)	(Mean Rank)	**sig. 2-tailed** *Mann Whitney Test*	
Parent Education		11.75	12.39	0.817	
Current job responsibility of parent		13.54	9.61	0.156	
Total Combined Family Income^b^		13.79	9.22	0.080	
***Executive Function Measures***				**sig. 1-tailed** *Independent samples t-test*	
DKEFS	Trail Making^a^	9.33±1.76	7.33±2.24	0.026	* +
	Verbal Fluency	10.80±2.51	8.17±3.56	0.016	* +
	Color-Word Interference	10.20±1.21	9.92±2.19	0.336	
WISC	Digit Span Backwards	9.80±2.36	10.81±2.52	0.151	
	Coding	11.13±1.99	9.17±2.41	0.013	*
***fMRI Shifting Task In-Scanner Performance***		%	%	**sig. 2-tailed** *Independent samples t-test*	
Univalent Rule Accuracy		95.97±0.09	95.41±0.08	0.865	
Bivalent Rule Accuracy		90.56±0.12	85.03±0.16	0.307	
Switching Accuracy		92.09±0.12	87.76±0.13	0.367	
Rule Representation		92.72±0.10	89.18±0.12	0.424	

+ significant with FDR Correction.

a one child did not finish all testing.

b Scale where 1 = $25 000–34 999, 2 = $35 000–49 999, 3 = $50 000–74 999, 4 = $75 000–99 999, 5 = $100 000+.

### Ethics Statement

This study was approved by the Boston Children's Hospital's Committee on Clinical Investigation (CCI). Written assent and informed consent were obtained from each child participant and guardian, respectively. All adult participants provided written informed consent.

### Measures

#### (i) Cognitive assessment

Adult participants completed the Delis-Kaplan Executive Function System as part of a larger study (DKEFS; [84]), and the subtests evaluating our hypotheses were analyzed here (Trail Making; Verbal Fluency; Color-Word Interference; Design Fluency). Children completed a matched subset of DKEFS subtests in order to maximize attention and avoid fatigue (Trail Making; Verbal Fluency; Color-Word Interference). Dependent variables included standardized outputs from each subtest.

- The Trail Making subtest assesses visual scanning, numeric and alphabetic sequencing, motor speed, and cognitive flexibility. Participants are timed on their ability to trace objects within a specified order when scrambled across a large sheet of paper, and corrected for errors throughout. The task includes five trials, (1) line tracing, (2) number tracing, (3) letter tracing, (4) number-letter switching, and (5) motor speed. The task of interest is a number-letter switching test in which the participant is required to draw straight lines to connect numbered and lettered circles in numerical and chronological order while switching between numbers and letters as quickly as possible. The output variable contrasted time to completion of the switching task to time required for the combined outcome of the two separate trials measuring number tracing and letter switching.
- The Verbal Fluency subtest contains three conditions that measure letter fluency, category fluency, and category switching fluency. Our output of interest compared achievement on letter fluency with category fluency. In letter fluency, participants were prompted with a single letter and asked to state as many words starting with that letter as possible within 60 seconds, excluding names of people, places, or numbers. In category fluency, participants were prompted with a category (e.g. boy's names, animals) and asked to name as many objects within the category of interest as possible within 60 seconds. Category switching fluency required participants to switch naming between two categories simultaneously. Responses were standardized based on the number of correct words named.
- The Color-Word Inference Test, based on the Stroop test [85], measures a participant's inhibition control by verbal naming of the printed ink color of a conflicting colored word as quickly and accurately as possible. This was contrasted with a pure color-naming task. The standardized output variable was derived from the contrast of time to completion for the inhibition task to the color-naming task.
- Adults additionally completed the Design Fluency subtest of the DKEFS. Design Fluency involves three subtests that require the participant to connect a set series of dots to make as many different designs possible within 60 seconds. Performance on the third task, creating designs while switching between empty and filled dots, was compared in musicians and non-musicians.

Working memory and processing speed were evaluated through the Digit Span Backwards and Coding subtests (respectively) of the Wechsler Abbreviated Intelligence Scale, 4th Edition (WAIS-IV; [86]) and Wechsler Intelligence Scale for Children IV (WISC-IV; [87]). The Digit Span subtest required participants to correctly echo a string of numbers orally in backwards order, presented with increasing length of digit span. Although Digit Span includes two subtests, Forward and Backward, Forward Digit Span is not generally regarded as a measure of EF and therefore was not included in the present analysis [22]. The Coding subtest asks participants to code as many specific symbols to corresponding numbers in randomized order as possible within 120 seconds.

In order to match general cognitive ability across groups, nonverbal IQ was tested in our children with the Kaufman Brief Intelligence Test (KBIT; [88]), and verbal and nonverbal IQ in adults with the Wechsler Abbreviated Scale of Intelligence (WASI; [89]). Based on our a priori hypotheses that musicians compared to non-musicians will show better performance on EF measures, independent-sample t-tests (one-tailed) were employed to compare performance on executive measures between musicians and non-musicians, corrected for multiple comparisons.

#### (ii) fMRI set-shifting task (Children only)

**Figure 1:**
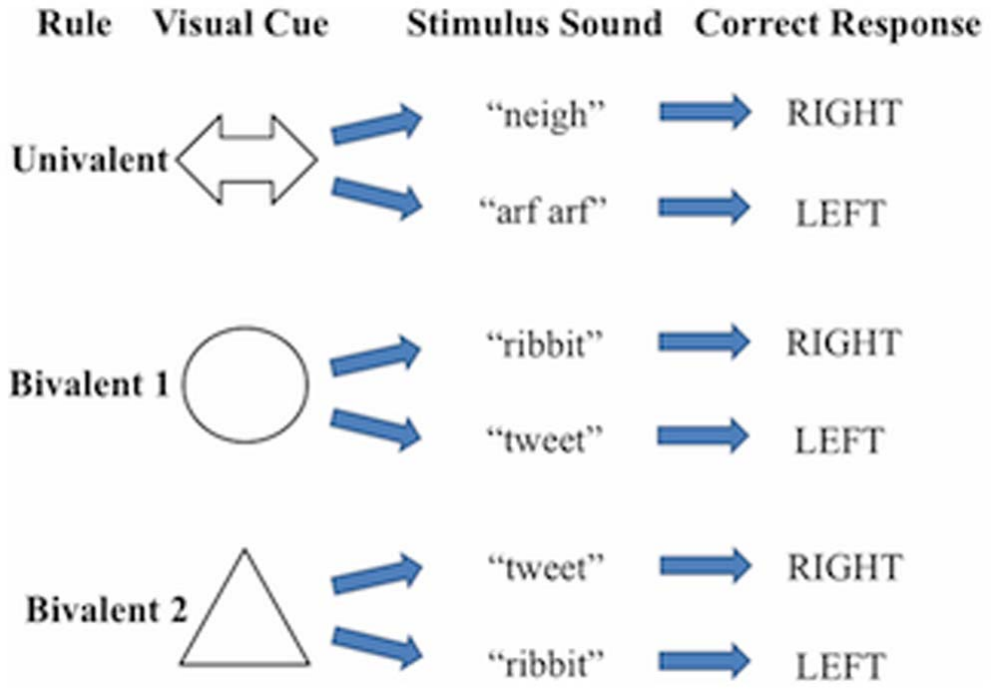
Cross-modal shifting task (fMRI). In each trial a cue [arrow; circle; or triangle] representing a rule was followed by a sound. Children responded with a left or right button press (arrow: horse  =  right; dog  =  left; circle: frog = right; bird = left; triangle: bird = right; frog = left). Critically, in one instance the rule consistently maps to single auditory stimuli (univalent rule) while in the latter two the auditory stimulus-response relationship changes with the visual cue (bivalent rules).

A multi-modal version of a traditional set-shifting task was developed (Figure 1) after Crone *et al*. [8] and implemented in the musically trained and untrained children. Auditory stimuli were incorporated in this task since musical training has shown specialization in the auditory domain [90] and since Pallesen *et al*. [79] observed differences in prefrontal and SMA areas during an auditory working memory task. Rules were indicated by visual cues (n = 3) followed by auditory stimuli to button presses (left, right), which included one univalent rule, where the auditory stimuli consistently mapped to left and right responses; and two bivalent rules where the sound alternately mapped a left or right response. Specifically, for the univalent rule condition, children would see an arrow followed by either the sound of a horse (“neigh”) or a dog (“arf arf”) 500 ms later. The task was then to press the right button for the horse and the left for the dog. In the bivalent rule condition, children would see either a circle or triangle, and 500 ms later hear either a frog sound (“ribbit”) or bird sound (“tweet”). If the circle was presented, the task was to press the right button for the frog and the left button for the bird, whereas if the triangle was presented, children were instructed to press the left button for the frog and the right button for the bird. Trials included a cue (1000 ms), break (500 ms) then auditory stimulus (2000 ms) followed by a crosshair until the subsequent trial commenced. Participants trained on 15 trials of each rule, then on a single session with all rules intermixed for 90 trials. In the fMRI task, each participant completed two sessions with 90 trials each (30 trials of each rule type: 30 with a univalent and 60 with bivalent rule conditions (30 for each bivalent rule 1 and 2), with sessions counterbalanced across participants and matched between musicians and non-musicians. Out of the 90 trials, approximately 12% were univalent rule repetitions (univalent rule trial → univalent rule trial); approximately 22% were univalent switches (switch from bivalent rule 1 trial → univalent rule trial and bivalent rule 2 trial → univalent rule trial), approximately 22% were bivalent repetitions (bivalent rule 1 trial → bivalent rule 1 trial and bivalent rule 2 trial → bivalent rule 2 trial), approximately 22% were bivalent switches (switch from univalent rule trial → bivalent rule 1 trial and univalent rule trial → bivalent rule 2 trial) and approximately 22% were bivalent reconfigurations (switch from bivalent rule 1 → bivalent rule 2 and vice versa).

Trial type and switch type were both randomized within each run using optseq (http://surfer.nmr.mgh.harvard.edu/optseq/). The use of optseq also maximized sensitivity to task-related blood flow by optimizing the duration and temporal jittering of each condition. In-scanner performance was qualified through the following outputs: univalent rule accuracy, bivalent rule accuracy, switching accuracy, and rule representation (the average accuracy across all conditions). Two fMRI runs were conducted on a Siemens 3 T Trio scanner in an event-related paradigm (255 images; 32-slices; interleaved ascending acquisition; 4 mm thick; 3×3×4 mm voxels; repetition time (TR) = 2000 ms, echo time (TE) = 30 ms, flip angle = 90°, FOV = 193×153×153 mm). Preprocessing and subsequent analyses were completed in FSL 4.1.4 (http://www.fmrib.ox.ac.uk/fsl/). Modeling was conducted with FEAT v5.98, and higher-level analysis with FLAME 1. Six initial images were discarded (field effects). Preprocessing included motion correction (MCFLIRT); slice-time correction; skull-stripping (BET); smoothing (4 mm FWHM kernel); temporal filtering (50 s high-pass filter); and linear registration (12 DOF; FLIRT) to the MNI152 T1 template. For artifact detection, images where mean signal drifted more than three standard deviations or participants moved more than 1.5 mm were modeled separately (http://web.mit.edu/swg/art/). In five cases (three controls, two musicians) 254 images were acquired and it was unclear whether stimulus onsets synchronized with the first or second scan due to hardware issues. The correct model was determined by generating the possible models and selecting the model with greatest Heschl's gyrus signal change (ROI analysis; Harvard-Oxford cortical atlas) for the contrast of all trial types > null. This contrast reflects the only time during the task when participants were presented auditory stimuli, such that the correct time course for these conditions would yield the greatest (audition-related) change in blood flow.

The following regressors were modeled: bivalent rule repetitions and switches, univalent rule repetitions and switches, and bivalent rule reconfigurations. Correct and incorrect trials were modeled separately, as were misses. Trials commenced with the visual cue and terminated at the end of the auditory stimulus (3.5 s). Each child's session level models were combined into fixed effects models; children were then combined in random effects analyses. Statistical inference was completed using Z (Gaussianised t) images, cluster thresholded (Z>2.3; *p* = 0.05 corrected). Rule representation was examined through the contrast of [all bivalent > all univalent rule trials], and task-switching effects through [bivalent switches and reconfigurations > univalent switches]. Independent two-sample t-tests were employed (*p* = 0.005 uncorrected) to examine differences in brain activation during these contrasts between the two groups of children. These regions' engagement in specific forms of rule representation/switching was further interrogated through Region of Interest (ROI) analyses, comparing rule switching (univalent, bivalent, bivalent reconfiguration) with univalent and bivalent rule repetitions. Bilateral ventrolateral prefrontal cortex (PFC), supplemental motor area (SMA), and superior parietal ROIs were defined through the contrast [all accurate rule trials > null; all participants] masked anatomically with the VLPFC, SMA, and superior parietal regions (defined through the WFU Pickatlas, Harvard-Oxford Cortical Atlas). Mean contrast of parameter estimate (COPE) values were extracted from contrasts of interest (rule representation and task-switching) in each participant and compared via one-tailed paired t-tests based on our strong a priori hypotheses.

## Results

### Behavioral Results in Adults

Independent t-tests (one-tailed, FDR corrected [91]) revealed that adults with musical training performed significantly higher than non-musicians on standardized measures of Verbal Fluency (*p* = 0.018), Design Fluency (*p* = 0.003), Backward Digit Span (*p* = 0.001), and a trend towards significance for Coding (*p* = 0.098). No differences in performance were found for Color-Word Interference (*p* = 0.160) or Trail Making (*p* = 0.423).

### Behavioral Results in Children

Musically trained children performed better than untrained children (independent t-tests, one-tailed, *p*<0.05, corrected for multiple comparisons) on Coding (*p* = 0.013), Verbal Fluency (*p* = 0.016) and Trail Making (*p* = 0.026). Standardized performance and behavioral characteristics of adults and children are outlined in Table 2. No significant difference in performance was observed for Color-Word Interference (*p* = 0.336) or Digit Span Backwards (*p* = 0.151).

### In-scanner behavioral results (Children only)

In-scanner performance revealed that both groups achieved high performance accuracy in rule representation and task-switching. Accordingly, no significant differences in behavioral scanner performance were observed for musically trained versus untrained children on univalent rule accuracy, bivalent rule accuracy, switching accuracy, or rule representation (see Table 2).

### fMRI Results (Children only)

**Figure 2:**
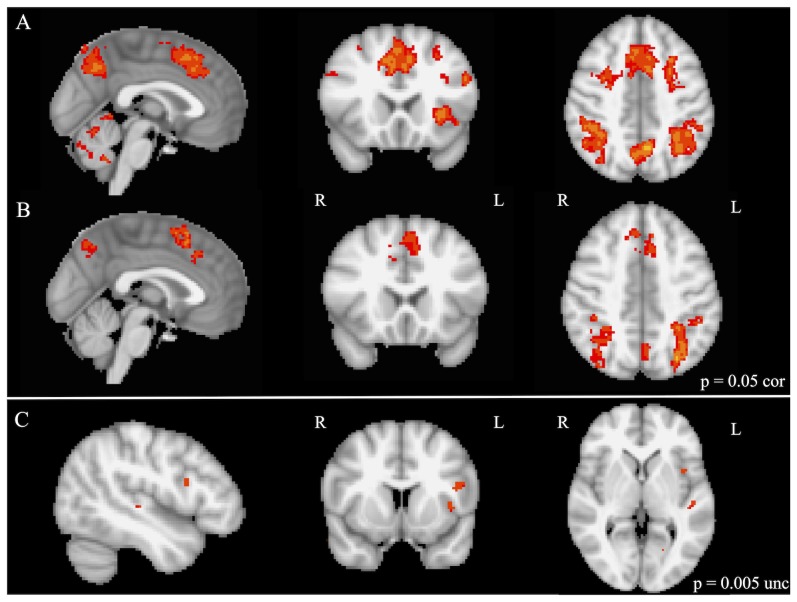
Whole brain activation during rule representation (all bivalent > all univalent rule trials) in (A) musically trained (p<0.05 corrected), (B) musically untrained (p = 0.05 corrected), and (C) two-sample comparison of musically trained over untrained children (p = 0.005 uncorrected). Note: activation is displayed with the FSL radiological convention.

**Figure 3:**
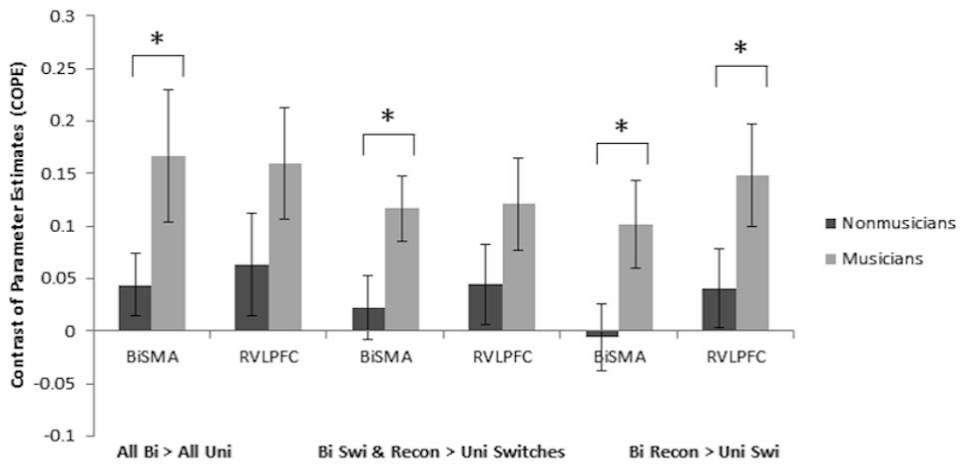
Mean contrast of parameter estimates (COPE) values extracted from the ROI analyses of musically trained compared with untrained children in bilateral SMA (BiSMA) and right VLPFC (RVLPFC) (* indicates significant at the p<0.05 threshold; for the rule representation contrast (bivalent > univalent rule trials)).

Whole brain analyses of rule representation (contrast: all bivalent > all univalent rule trials) demonstrated significant activation for both groups in several brain regions including the SMA/paracingulate cortex and VLPFC bilaterally for the musically trained group only (Figure 2). Further activation was apparent within regions including bilateral superior parietal cortex (angular and supramarginal gyri), insula and cerebellum (see Table 3). An independent two-sample t-test (*p* = 0.005 uncorrected) revealed significantly greater activation for musically trained compared to untrained children in the left VLPFC and left Heschl's gyrus (as shown in Figure 2 and Table 3). The opposite comparison of musically untrained children over trained children resulted in no cortical activation. To account for the uncorrected threshold at the whole brain level and further explore our a priori hypotheses, ROI analysis was then employed to evaluate the activation within our specific regions of interest. Extraction of the contrast of parameter estimates (COPE) during rule representation (bivalent > univalent rule trials) for each child within our specified ROIs (VLPFC, SMA, superior parietal cortex) revealed significant differences in bilateral SMA between the groups (*p* = 0.048), with the musically trained children demonstrating greater SMA activation (see Figure 3). No significant differences in VLPFC or superior parietal ROIs were found between musically trained and untrained children for this contrast (rule representation).

**Table 3:** Whole brain activation for musically trained and untrained children separately (one sample t-test) and two-sample t-test comparison (musically trained > untrained) during rule representation (contrast: all bivalent > all univalent rule trials).

*Musically Trained Children (p<0.05 cluster threshold >50 voxels)*							
		Coordinates					
Voxels	Maximum (Z)	x	y	z	Cerebrum	BA	Region
1619	3.82	8	22	40	R	8	Middle/Superior frontal gyrus (pre-SMA/SMA)
1355	3.72	−34	−60	44	L	7	Lateral Occipital Cortex/Superior Parietal Cortex
1253	3.92	42	−42	48	R	40	Supramarginal Gyrus (Inferior Parietal Lobule)
933	3.55	−46	12	30	L	9	Middle Frontal Gyrus (DLPFC)
854	4.03	−6	−58	44	L	7	Precuneous
645	3.71	−34	2	56	L	6	Middle Frontal Gyrus (pre-SMA/SMA)
590	3.35	8	−80	−26	R		Cerebellum
456	3.53	−30	−60	−34	L		Cerebellum
409	3.39	32	2	46	R	6	Middle Frontal Gyrus (pre-SMA/SMA)
396	3.22	40	30	24	R	46	Middle Frontal Gyrus (VLPFC)
308	3.77	−30	24	−6	L	47	Inferior Frontal Gyrus (VLPFC)

*Musically untrained children (p<0.05 cluster threshold >50 voxels)*							
		Coordinates					
Voxels	Maximum (Z)	x	y	z	Cerebrum	BA	Region
1531	4.12	−30	−70	40	L	19	Lateral Occipital/Superior Parietal Cortex (Precuneous)
936	3.53	34	−60	66	L		Superior Lateral Occipital Cortex
514	3.77	2	18	50	R	8	Middle/Superior Frontal Gyrus (pre-SMA/SMA)

*Musically Trained > Untrained Children (p<0.005 uncorrected)*							
		Coordinates					
Voxels	Maximum (Z)	x	y	z	Cerebrum	BA	Region
24	2.98	24	78	63	L	47	Inferior Frontal Gyrus (VLPFC)
14	2.9	24	78	39	L	44	Inferior Frontal Gyrus/Frontal Operculum
11	3	21	30	36	L	41	Heschl's Gyrus/Planum Temporale

Coordinates in MNI space, gray matter activations significant at p<0.05 with a cluster threshold >50 voxels for musically trained and untrained groups separately; p<0.005 uncorrected threshold for the two-sample t-test.

**Figure 4:**
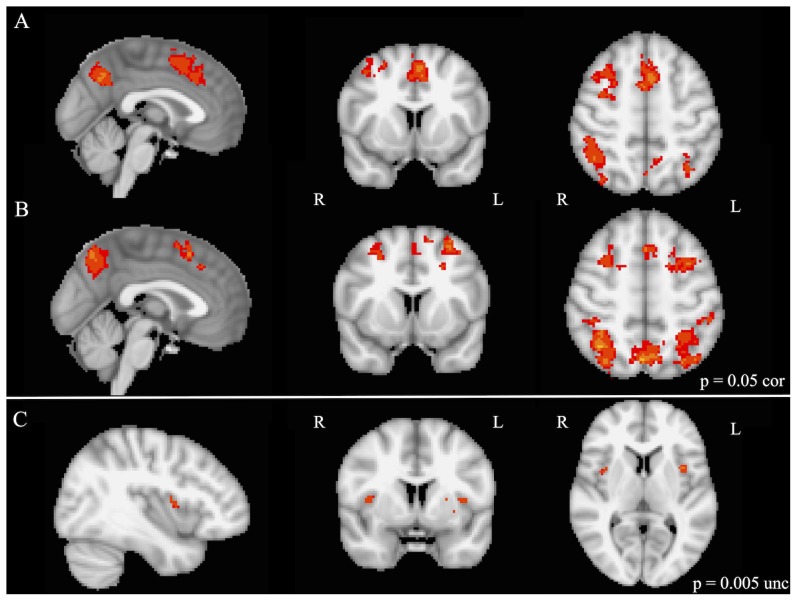
Whole brain activation during task-switching (bivalent switches and reconfigurations > univalent switches) in (A) musically trained (p<0.05 corrected), (B) musically untrained (p = 0.05 corrected), and (C) two-sample comparison of musically trained over untrained children (p = 0.005 uncorrected). Note: activation is displayed with the FSL radiological convention.

Whole brain analysis of task-switching (contrast: bivalent switches and reconfigurations > univalent switches) revealed a similar pattern of activation in bilateral SMA, VLPFC, and superior parietal regions in both groups of children (Figure 4, Table 4). Two-sample t-test comparisons (*p* = 0.005 uncorrected) of musically trained over untrained children demonstrated greater activation in bilateral prefrontal regions, specifically VLPFC (Figure 4, Table 4). Enhanced activation in the right supramarginal gyrus was found in musically trained over untrained children as well. Conversely, two-sample t-test comparison of musically untrained over trained children identified greater left superior parietal activation in untrained children. ROI analysis for the task-switching contrast (bivalent rule switches and reconfigurations > univalent switches) revealed that musically trained children demonstrated greater activation in bilateral SMA (*p* = 0.021) compared to untrained children, with the effect being likely driven by the greater complexity of bivalent rule reconfigurations (bivalent rule reconfigurations > univalent switches; *p* = 0.027; Figure 3). No significant differences were found bilaterally in the VLPFC between musically trained and untrained children; however, musically trained children demonstrated significantly greater activation specifically within the right VLPFC when switching to more complex rather than simple rule representations (bivalent rule reconfiguration > univalent switches; *p* = 0.046; Figure 3). No significant differences in the superior parietal ROIs were found between musically trained and untrained children during task-switching.

**Table 4:** Whole brain activation for musically trained and untrained children separately (one sample t-test) and two-sample t-test comparison (musically trained > untrained) during task-switching (contrast: bivalent switches and reconfigurations > univalent switches).

*Musically Trained Children (p<0.05 cluster threshold > 50 voxels)*							
		Coordinates					
Voxels	Maximum (Z)	x	y	z	Cerebrum	BA	Region
1171	3.64	49	96	99	R	8	Middle/Superior Frontal Gyrus (pre-SMA/SMA)
988	3.63	69	−24	99	R	7	Lateral Occipital/Superior Parietal Cortex (Precuneous, Angular Gyrus)
532	4.29	42	−24	102	L	19	Lateral Occipital/Superior Parietal Cortex (Precuneous)
485	3	61	66	102	R	8	Middle/Superior Frontal Gyrus (pre-SMA/SMA)
384	3.58	29	−27	102	L	7	Lateral Occipital/Superior Parietal Cortex (Precuneous, Angular Gyrus)

*Musically untrained children (p<0.05 cluster threshold > 50 voxels)*							
		Coordinates					
Voxels	Maximum (Z)	x	y	z	Cerebrum	BA	Region
1367	4.32	28	−36	96	L	7	Lateral Occipital/Superior Parietal Cortex (Precuneous, Angular Gyrus)
1273	4.31	62	−14	114	R	7	Lateral Occipital/Superior Parietal Cortex (Angular Gyrus)
662	4.26	47	−39	114	R	7	Precuneous Cortex
533	3.67	29	72	114	L	8	Middle/Superior Frontal Gyrus (pre-SMA/SMA)
283	3.62	59	72	108	R	8	Middle/Superior Frontal Gyrus (pre-SMA/SMA)
274	3.57	42	90	108	L	8	Superior Frontal Gyrus

*Musically Trained > Untrained Children (p<0.005 uncorrected)*							
		Coordinates					
Voxels	Maximum (Z)	x	y	z	Cerebrum	BA	Region
52	3.56	27	75	45	L	44	Inferior Frontal Gyrus/Frontal Operculum (VLPFC)
25	2.91	64	72	51	R	44	Inferior Frontal Gyrus/Frontal Operculum (VLPFC)
21	2.95	75	27	75	R	40	Supramarginal Gyrus (Inferior Parietal Lobule)

Coordinates in MNI space, gray matter activations significant at p<0.05 with a cluster threshold >50 voxels for musically trained and untrained groups separately; p<0.005 uncorrected threshold for the two-sample t-test.

## Discussion

Our study employed strict participant inclusion criteria and utilized standardized psychometric measures to clarify the mixed findings to date on the relationship between musical training and EF abilities. We further explored the associated neural correlates of task-switching in musically trained over untrained children through fMRI. Overall, adult musicians and musically trained children showed heightened performance on several but not all constructs of EF, and children further demonstrated enhanced brain activation in traditional EF regions during a task-switching paradigm. Behavioral differences between adult musicians and non-musicians were observed for measures of cognitive flexibility (such as Verbal Fluency, Design Fluency, Trail Making) and working memory. Musically trained and untrained children also significantly differed on measures of cognitive flexibility (Verbal Fluency and Trail Making) and processing speed as well. The investigation of the neural correlates of rule representation and task-switching revealed greater activation in the SMA and right VLFPC for musically trained as compared to untrained children. These results support the working hypothesis that musical training may promote the development and maintenance of EF, which could mediate the previous reported links between musical training and heightened academic achievement, though our cross-sectional study presently cannot address whether prior EF abilities may have promoted the development of musical training.

The connection between musical skill and specific components of EF is conceivable, given the demands of sustained attention, goal-directed behavior, and cognitive flexibility that are involved in musical training. Our results are in line with some prior evidence of a connection between musical training and certain EF constructs, particularly the previously reported association between several EF constructs in children with varying intensities of musical training [49] and the observed improvement in cognitive flexibility and improved working memory following piano intervention in elderly adults [82]. However, the specific components of EF we have observed to relate to musical training are somewhat inconsistent with other prior evidence. For instance, we did not observe previously reported differences in inhibition [49], [80], [81]. Discrepancies between our present findings and those of prior studied may be due to differences in subject selection criteria (e.g. careful screening for no musical training in controls as done in our study, obtaining data on the subject's socioeconomic (SES) background in 29/30 adults and 19/27 children and carefully matching the groups based on the obtained SES info), differences in EF measures included, or in sample size. Previously, a music-based computerized intervention demonstrated direct improvements on an inhibition task in 4–6 year old children [83]. This is inconsistent with our results in school-age children and adults but may be explained by a developmental trajectory effect for the cognitive construct inhibition. It may also be due to differences in the employed measures of inhibition between the two studies. While our study employed standardized measures of inhibition within a standardized EF battery, Moreno and colleagues employed a computerized inhibition task. Furthermore, it may be possible that the inconsistent findings can be explained by our relatively low sample size for behavioral studies. The effects of musical training on inhibition could, for example, be smaller than the effect of musical training on cognitive flexibility and our study may therefore lack the power to detect this effect. Future studies are needed to closer examine the interaction between musical training and the development of inhibition in early childhood.

Interestingly, significant differences in processing speed were only observed between musically trained and untrained children. The developmental trajectory of processing speed has been described to begin in childhood and continue until adolescence [24]. Therefore, it is possible that no differences in processing speed were observed between our adult musicians and non-musicians since processing speed has reached a performance plateau in this age range, whereas we have captured this ability at a time of (rapid) development in our children. Our assessment of working memory (Digit Span Backwards) resulted in significant differences between musicians and non-musicians in adults, but no differences in the child age range could be observed. Considering that a similar developmental trajectory has been described for working memory as for processing speed [21], [22], [25], it remains unclear why we only observe differences in adults. Various studies have explored an association between working memory and music training, and children and adults who have received music training have shown enhanced performance over non-musician controls on measures of auditory and visual working memory, such as forward and backward digit span (e.g. [74], [75], [76], [78], [79]), consistent with our findings in adults. In addition, significantly higher scores on digit span for musically trained children compared to control children have been previously reported and digit span scores further correlated with IQ [47]. However, Schellenberg (2011) examined the potential mediating effect of executive functioning on IQ, and therefore the groups were not matched for IQ. In our study, the adult sample was matched for IQ and significant differences in Digit Span were observed in this group. Interestingly, one prior study demonstrated that early-trained adult musicians (started before age seven) performed better on a rhythmic task than late-trained adult musicians (who started after age seven), which correlated with auditory working memory ability when otherwise groups did not differ in cognitive abilities [92]. Overall, the discrepancy between these studies suggests either a developmental effect or an effect of total duration of musical experience on working memory abilities, which should be examined more closely in future studies.

Only one previous study did not observe any differences between intensive musical training and executive function [47] but also implemented non-standardized methods for assessing EF skills. Contrary to this study, our investigation implemented standardized measures for EF evaluation, which may have increased the sensitivity to detect an effect. Additionally, Schellenberg (2011) suggested that individuals with high IQ might be more likely to pursue musical training than lower-performing peers, which could lead to biased results. Carefully matched IQ and socioeconomic status (education and income) between intensively trained musicians and absolute non-musicians in our sample allowed us to evaluate the relation between musical training and EF without these previous suggested confounding factors. While previous research has linked IQ and EF [93], group-matched IQ was an important factor in this study to determine the effect of musical training on EF to avoid potential confounds due to higher IQ in our musicians. Nonetheless, it remains unknown whether the observed association in musicians is due to primarily underlying strengths in cognitive flexibility, working memory, processing speed or a combination of these three and further, disentangling these constructs in relation to musical training will prove to be a challenge considering they are highly correlated [93], [94].

As for our neuroimaging evidence, we evaluated rule representation and task-switching in our sample of children using a multi-modal traditional EF task. Rule representation (contrast: bivalent > univalent rule trials) and task-switching (contrast: bivalent switches and reconfigurations > univalent switches) was associated with an activation increase within the VLPFC, SMA, and superior parietal cortex in all children regardless of musical training. This is consistent with previous results utilizing a similar task in adults and children [8]. For rule representation, musically trained children demonstrated significantly greater activation within the left VLPFC in whole brain comparison of the groups and bilateral SMA, as revealed through ROI analysis. No significant differences in parietal activation were found based on musical training during rule representation in either the whole brain or ROI analyses. Interestingly, enhanced activation was found in the left Heschl's gyrus in musically trained over untrained children during rule representation in the whole brain analysis, a brain region previously shown to be important for music processing and recruited more so by musicians than non-musicians during auditory tasks [72], [73], [95], [96], [97], [98].

As for activation differences based on musical training during task-switching, musically trained children demonstrated enhanced activation in the bilateral VLPFC in the whole brain two-sample comparison over untrained children. Although no significant differences in the superior parietal ROIs were found between groups, untrained children demonstrated more activation in the left superior parietal regions over musically trained children at the whole-brain level. This finding suggests that children with versus without musical training differentially recruit specific brain regions during task-switching. In particular, untrained children appear to recruit parietal regions within a network of activation that is typical for task-switching [9], whereas musically trained children rely significantly more on frontal regions during this task. ROI analysis additionally revealed significantly more activation in the bilateral SMA during task-switching in addition to rule representation for musically trained children over untrained children. In fact, greater activation in SMA with rule complexity was even more prominent in the more cognitively effortful switching tasks (contrast: bivalent rule reconfigurations > univalent switches). A developmental study in 8–25 year olds using a similar task could show that children, adolescents and adults engaged pre-SMA/SMA for task-switching, but that children aged 8–12 years additionally recruited the pre-SMA/SMA for rule representation [9]. This is consistent in our sample, since both groups engaged pre-SMA and SMA during rule representation and task-switching. Our results further suggest that the SMA is more engaged in children who are intensively trained musically, which is in line with the findings reported by Pallesen and colleagues [79] who examined working memory of musical sounds in musicians and non-musicians. Enhanced activation in musicians compared to non-musicians was reported in neuronal networks that sustain attention and cognitive control, including the supplementary motor area. Furthermore, the relationship between task performance and activation values was stronger in musicians than in non-musicians, especially during the highest working memory loads. We also observed enhanced activation of the SMA in our musically trained children and activation increase with rule complexity was more prominent in the more cognitively effortful switching tasks.

Interestingly, musically trained and untrained children both showed high performance accuracy on the neuroimaging task and no significant behavioral differences were observed. It may be possible that we did not find more robust activation differences in other regions, such as the parietal areas, due to this strong behavioral performance in both musicians and non-musicians. An alternative explanation to no significant activation differences in parietal regions could be that the effects of musical training do not enhance the aspects of executive functioning that are represented by parietal areas, but instead specifically engage prefrontal regions of the brain. No findings in other brain regions supports the evidence that distinct neural components are involved in task-switching [8], [9], and that musical training appears to selectively enhance frontal activation patterns during this task. Heightened engagement of pre-SMA and SMA has been shown in professional musicians as compared to non-musicians during various tasks including motor planning but also specific elements of musical engagement such as anticipation, timing, improvisational flexibility, and rhythmic demands or musical imagery [99], [100], [101]. For example, the neural representation of anticipation and execution of musical events has been evaluated in professional musicians by investigating activation patterns during oral rehearsal of music, and the SMA has been identified as a key area involved in the oral representation of percussion music [102]. Another fMRI study revealed that jazz musicians demonstrated a neural network for judged improvisations involving the SMA, frontal operculum, and anterior insula [103], suggesting that the SMA is involved in the detection of spontaneous musical performances. Overall, neuroimaging evidence points toward the involvement of SMA regions during lower- and higher-order features of musical performance. Based on the existing behavioral and neuroimaging evidence and results from our current study, one could hypothesize that musical training may reinforce SMA activity and its integration into the EF brain network which may, in turn, lead to improved behavioral EF skills, but further studies have to clarify this.

All children exhibited activation in the VLPFC for rule representation (contrast: bivariate > univariate rule trials). We further observed greater activation for musically trained as compared to untrained children in the right VLPFC during rule representation in the whole brain findings and for more complex rather than simple rule representations (bivariate rule reconfiguration > univariate switches) in the ROI analysis. This is in line with the study reported by Pallesen and colleagues [73], who also showed that working memory load-dependent activations in VLPFC during an auditory working memory task were stronger in adult musicians compared to non-musicians. Several previous studies have shown activation within the involvement of VLPFC in non-musicians and musicians for various music tasks (for a review, see [104]), such as tapping to the beat of musical rhythm [105], perception/judgment of irregular chords [96], acquired conditional associative memory for musical stimuli [106], and mental reversal of imagined melodies [107]. Interestingly, Koelsch and colleagues [96] reported heightened activation in adults compared to children in left prefrontal areas during the perception/judgment of irregular chords, which is in line with previous studies describing the developmental trajectory of rule representation [9]. Furthermore, it has been reported that rule representation and rule switching follow separate developmental trajectories [9] and the role of VLPFC in the developmental stages of cognitive flexibility are still debated. Examination of the adults' brain activation was beyond the scope of the present study; future work is needed to clarify whether differences can be observed in adult musicians and non-musicians during rule representation and task-switching. Thus, further studies with a variety of experimental tasks are needed to examine the possible influence of musical training on brain regions mediating executive functioning skills during development.

Undoubtedly, the connection between musical training and cognitive ability is highly complex, as previously argued by Schellenberg [47]. The present study precludes determination of whether enhanced EF abilities in musicians are a direct consequence of musical training or rather the predisposition to study music. Specifically, Schellenberg (2011) has called to question whether children with higher cognitive skills are more likely to succeed with musical training, and thus characterize the individuals who continue long-term training in music. The argument has been presented that children with higher IQ's represent the musician group compared to their non-musical peers with lower IQ's, however, this claim has been debated due to the methodological limitations of the study [48], [50]. Although children and adults with musical training in our sample have demonstrated superior performance on a number of specific EF constructs over non-musicians, a number of considerations present as to the origin of this link and the other contributing factors that may be present. It remains unknown whether the connection between musical training and EF abilities is the same for every individual, or how much this relation depends on individual cognitive dispositions and varying musical experiences. In the present study, we obtained reports of type and intensity of training, documenting individual practice versus engagement within a group ensemble, but were not able to account for all of these potential differences in experience in the present analysis due to our sample size. While our findings show that with carefully matched achievement between groups we find enhanced EF abilities in musicians, this study cannot address the causal nature of this connection. Future studies need to examine the influence of musical training on EF abilities longitudinally through random assignment in order to determine the directionality of this connection and to examine the individual contributions of these different components of EF.

Overall, we conclude that children and adults with extensive musical training show enhanced performance on a number of EF constructs compared to non-musicians, especially for cognitive flexibility, working memory, and processing speed. Investigation of the neural correlates of rule representation and task-switching further revealed heightened activation in bilateral SMA and left VLPFC for musically trained as compared to untrained children through direct whole brain comparison and ROI analysis. Thus, our results support the working hypothesis that executive functioning may be one of the mechanisms mediating the often reported link between musical training and heightened academic skills, as EF skills and academic skills are highly correlated. However, more longitudinal studies and interventions are needed in order to examine a possible causal relationship between musical training, EF skills and academic achievement. Furthermore, behavioral and neural developmental trajectories for various EF skills need to be examined for musicians as compared to non-musicians. Nevertheless, future studies examining cognitive and academic skills between musicians and non-musicians should control for various components of EF. Likewise, it is important to consider that replacing music programs with reading or math instruction in our nation's school curricula in order to boost standardized test scores may actually lead to deficient skills in other cognitive areas.
