# Correction: Behavioral and Neural Correlates of Executive Functioning in Musicians and Non-Musicians

**DOI:** 10.1371/journal.pone.0137930

**Published:** 2015-09-02

**Authors:** Jennifer Zuk, Christopher Benjamin, Arnold Kenyon, Nadine Gaab

There is an error in Table 1. The Mean ± SD of ‘Duration of musical training (years)’ for ‘Adult Musicians’ is incorrect. Please see the correct Table 1 here.

**Table 1:** Group characteristics of musical experience in adult musicians and musically trained children.

	Mean ± SD
**Adult Musicians (n = 15)**	
***Group Characteristics***	
Age at onset of musical training (years)	5.73 ± 1.62
Intensity of practice time/week (hours)	21.87 ± 11.49
Duration of musical training (years)	18.93 ± 3.13
***Type of Musical Instrument***	*Number of adults*
Piano	6
Strings	5
Woodwinds	1
Brass	2
Harp	1
**Musically Trained Children (n = 15)**	
***Group Characteristics***	
Age at onset of musical training (years)	5.86 ± 1.41
Intensity of practice time/week (hours)	3.74 ± 2.63
Duration of musical training (years)	5.2 ± 1.33
***Type of Musical Instrument***	*Number of children*
Piano	5
Strings	5
Woodwinds	2
Guitar	1
Percussion	2
