# Correction: Behavioral and Neural Correlates of Executive Functioning in Musicians and Non-Musicians

**DOI:** 10.1371/journal.pone.0191394

**Published:** 2018-01-11

**Authors:** Jennifer Zuk, Christopher Benjamin, Arnold Kenyon, Nadine Gaab

In Tables 3 and 4, there are incorrect values. Please see the corrected Tables 3 and 4 here.

**Table 3:** Whole brain activation for musically trained and untrained children separately (one sample t-test) and two-sample t-test comparison (musically trained > untrained) during rule representation (contrast: all bivalent > all univalent rule trials). Coordinates in MNI space, gray matter activations significant at p<0.05 with a cluster threshold > 50 voxels for musically trained and untrained groups separately; p<0.005 uncorrected threshold for the two-sample t-test.

***Musically Trained Children (p<0*.*05 cluster threshold > 50 voxels)***							
		**Coordinates**					
**Voxels**	**Maximum (Z)**	**x**	**y**	**z**	**Cerebrum**	**BA**	**Region**
1619	3.82	8	22	40	R	8	Middle/Superior frontal gyrus (pre-SMA/SMA)
1355	3.72	-34	-60	44	L	7	Lateral Occipital Cortex/Superior Parietal Cortex
1253	3.92	42	-42	48	R	40	Supramarginal Gyrus (Inferior Parietal Lobule)
933	3.55	-46	12	30	L	9	Middle Frontal Gyrus (DLPFC)
854	4.03	-6	-58	44	L	7	Precuneous
645	3.71	-34	2	56	L	6	Middle Frontal Gyrus (pre-SMA/SMA)
590	3.35	8	-80	-26	R		Cerebellum
456	3.53	-30	-60	-34	L		Cerebellum
409	3.39	32	2	46	R	6	Middle Frontal Gyrus (pre-SMA/SMA)
396	3.22	40	30	24	R	46	Middle Frontal Gyrus (VLPFC)
308	3.77	-30	24	-6	L	47	Inferior Frontal Gyrus (VLPFC)
***Musically untrained children (p<0*.*05 cluster threshold > 50 voxels)***							
		**Coordinates**					
**Voxels**	**Maximum (Z)**	**x**	**y**	**z**	**Cerebrum**	**BA**	**Region**
1531	4.12	-30	-70	40	L	19	Lateral Occipital/Superior Parietal Cortex (Precuneous)
936	3.53	34	-60	66	R		Superior Lateral Occipital Cortex
514	3.77	2	18	50	R	8	Middle/Superior Frontal Gyrus (pre-SMA/SMA)
***Musically Trained > Untrained Children (p<0*.*005 uncorrected)***							
		**Coordinates**					
**Voxels**	**Maximum (Z)**	**x**	**y**	**z**	**Cerebrum**	**BA**	**Region**
24	2.98	-42	10	18	L	47	Inferior Frontal Gyrus (VLPFC)
14	2.9	-42	10	2	L	44	Inferior Frontal Gyrus/Frontal Operculum
11	3	-48	-22	0	L	41	Heschl’s Gyrus/Planum Temporale

**Table 4:** Whole brain activation for musically trained and untrained children separately (one sample t-test) and two-sample t-test comparison (musically trained > untrained) during task-switching (contrast: bivalent switches and reconfigurations > univalent switches). Coordinates in MNI space, gray matter activations significant at p<0.05 with a cluster threshold > 50 voxels for musically trained and untrained groups separately; p<0.005 uncorrected threshold for the two-sample t-test.

***Musically Trained Children (p<0*.*05 cluster threshold > 50 voxels)***							
		**Coordinates**					
**Voxels**	**Maximum (Z)**	**x**	**y**	**z**	**Cerebrum**	**BA**	**Region**
1171	3.64	8	22	42	R	8	Middle/Superior Frontal Gyrus (pre-SMA/SMA)
988	3.63	48	-58	42	R	7	Lateral Occipital/Superior Parietal Cortex (Precuneous, Angular Gyrus)
532	4.29	-6	-58	44	L	19	Lateral Occipital/Superior Parietal Cortex (Precuneous)
485	3	32	2	44	R	8	Middle/Superior Frontal Gyrus (pre-SMA/SMA)
384	3.58	-32	-60	44	L	7	Lateral Occipital/Superior Parietal Cortex (Precuneous, Angular Gyrus)
***Musically untrained children (p<0*.*05 cluster threshold > 50 voxels)***							
		**Coordinates**					
**Voxels**	**Maximum (Z)**	**x**	**y**	**z**	**Cerebrum**	**BA**	**Region**
1367	4.32	-34	-66	40	L	7	Lateral Occipital/Superior Parietal Cortex (Precuneous, Angular Gyrus)
1273	4.31	34	-58	52	R	7	Lateral Occipital/Superior Parietal Cortex (Angular Gyrus)
662	4.26	4	-68	52	R	7	Precuneous Cortex
533	3.67	-32	6	52	L	8	Middle/Superior Frontal Gyrus (pre-SMA/SMA)
283	3.62	28	6	48	R	8	Middle/Superior Frontal Gyrus (pre-SMA/SMA)
274	3.57	-6	18	48	L	8	Superior Frontal Gyrus
***Musically Trained > Untrained Children (p<0*.*005 uncorrected)***							
		**Coordinates**					
**Voxels**	**Maximum (Z)**	**x**	**y**	**z**	**Cerebrum**	**BA**	**Region**
52	3.56	-36	8	6	L	44	Inferior Frontal Gyrus/Frontal Operculum (VLPFC)
25	2.91	38	6	10	R	44	Inferior Frontal Gyrus/Frontal Operculum (VLPFC)
21	2.95	60	-24	26	R	40	Supramarginal Gyrus (Inferior Parietal Lobule)
